# Memory Regulatory T cells Increase Only In Inflammatory Phase of Chronic Hepatitis B Infection and Related to Galectin-9/Tim-3 interaction

**DOI:** 10.1038/s41598-017-15527-x

**Published:** 2017-11-10

**Authors:** Ching-Chih Hu, Wen-Juei Jeng, Yi-Cheng Chen, Jian-He Fang, Chien-Hao Huang, Wei Teng, Yi-Chung Hsieh, Yung-Chang Lin, Rong-Nan Chien, I-Shyan Sheen, Chun-Yen Lin

**Affiliations:** 1 Graduate Institute of Clinical Medical Science, College of Medicine, Chang Gung University, Kweishan, Taoyuan Taiwan; 2 Division of hepatology, Department of Hepatogastroenterology, Chang Gung Memorial Hospital, Keelung, Taiwan; 3 Devision of Hepatology, Department of Hepatogastroenterology, Linkou Medical Center, Chang Gung Memorial Hospital, Tayouan, Taiwan; 4 Department of Hematology-Oncology, Linkou Medical Center, Chang Gung Memorial Hospital, Kweishan, Tayouan Taiwan

## Abstract

CD4^+^Foxp3^+^ regulatory T cells (Tregs) are the main immune suppressors with subpopulation of inflamed-tissue related memory Tregs (mTregs) and non-related resting Treg (rTregs). Previously, Treg was proposed to be the cause of chronicity of hepatitis B virus (HBV) infection but with controversies. We then investigated the role of mTregs in distinct immune phases of chronic HBV infection, especially the non-inflammatory versus inflammatory phases. It was found mTregs but not rTregs increased only in the inflammatory phase and correlated with serum alanine aminotransferase (ALT) level. These mTregs accumulated in the inflamed liver, expressed significantly higher Tim-3, CCR4, CCR5 and fewer CCR7, and possessed potent suppressive function. These mTregs mainly originated from natural Tregs because of high Helios expression. Hierarchical clustering analysis showed higher frequency of mTreg was concordant with higher serum ALT and galectin-9 levels. Furthermore, galectin-9 could expand mTregs through galectin-9/Tim-3 interaction. In conclusion, increased mTregs are found only in inflammatory phase of chronic HBV infection. Galectin-9, associated with liver inflammation, contributes to the expansion of mTregs through galectin-9/Tim-3 interaction. Therefore, this expansion of mTregs only reflects as an immune regulatory mechanism to limit the on-going liver damages rather than the cause of chronicity of HBV infection.

## Introduction

Chronic HBV infection is a serious global health problem, affecting more than 350 million people worldwide, with potential adverse sequelae like cirrhosis, liver failure and hepatocellular carcinoma^1,2^. The natural course of chronic HBV infection could be categorized into four distinct phases: the immune tolerant (IT); immune clearance (IC); low replicative (LR); and hepatitis B e antigen (HBeAg)-negative hepatitis (ENH) phases, with different clinical characteristics of viral replication and liver disease progression^3^. The IT phase is characterized by high serum level of HBV-DNA and hepatitis B s antigen (HBsAg) level but little liver inflammation. On the contrary, rapid liver fibrosis progression is only observed during the IC and ENH disease phases that are characterized with significant liver inflammation^3,4^. The traditional explanation for the IT phase is lack of efficient HBV-specific CD8^+^ and CD4^+^T cell responses and resurgence of these T cell responses initiates the IC phase^5,6^. However, recent new observations about increased transcript levels of interferon-stimulated genes^7^ and the less compromised HBV-specific T-cell responses in IT phase than in IC/ENH phases^8^ have urged a new immune perspective on these four distinct phases. Therefore, a new notion of non-inflammatory (IT and LR) and inflammatory (IC and ENH) phases of chronic HBV infection emphasizing the liver inflammation was proposed^6^.

On the other hand, regulatory T cells (Tregs) are a specialized T cell population with the ability to suppress immune responses and maintain immunological tolerance^9^. Previous studies had shown an increased frequency of Tregs in patients with chronic hepatitis B (CHB)^10,11^ and the depletion of Tregs could increase CD8^+^T cells activity^10–12^. In addition, the increase of Tregs was associated with reduction of treatment response to pegylated interferon-α and increased risk of hepatocellular carcinoma^12^. Furthermore, several researchers also proposed these Tregs might be the cause of the chronicity of HBV infection^11,13,14^, though others believed it only reflected the host’s attempt to protect itself against immune-mediated pathology^15^. However, almost all the studies were focused on patients with chronic HBV infection in the phases of IC and/or ENH. The role of Tregs in the natural history of chronic HBV infection had not been fully explored.

Recently, Tregs were further divided into resting Tregs (rTregs) and memory Tregs (mTregs) in mouse model. The mTregs could migrate into inflamed tissue to mitigate tissue damage during the heightened responses of pro-inflammatory memory cells^16^. On the other hand, human Tregs had been grouped into three phenotypic and functional distinct subpopulations according to the expression of CD45RA and FoxP3: CD45RA^+^Foxp3^lo^ Tregs, CD45RA^−^Foxp3^hi^ Tregs, and CD45RA^−^Foxp3^lo^ Tregs^17^. The last one, lacking the ability to suppress T cell proliferation, was not a suppressor. The first two were potent suppressors. It had been proposed that the CD45RA^+^Foxp3^lo^ Tregs were corresponding to human counterpart of rTregs and the CD45RA^−^Foxp3^hi^ Tregs were corresponding to human counterpart of mTregs in mouse^16^. As for the human disease situation, it had been demonstrated that the rTregs increased but mTregs decreased in patients with systemic lupus erythromatosus^17^. On the contrary, mTregs increased in patients with sarcoidosis^17^, colon cancer^18–20^ and hepatocellular carcinoma^18^. Furthermore, these mTregs, possessing pro-inflammatory chemokine receptors, could migrate to the inflammed tissue and inhibit the immune responses in inflamed tissue^18,19^.

We therefore investigate the role of Tregs in these four distinct phases of chronic HBV infection in this study, especially considering the non-inflammatory versus inflammatory phases. In addition, we also study the different Tregs including rTregs and mTregs in this scenario.

## Results

### Baseline demographic and clinical characteristics of the study population

There were totally 124 chronically HBV infected patients and 38 healthy volunteers enrolled into the study. The baseline characteristics of the 162 patients were listed in Table 1. Compatible with the natural history of chronic HBV infection, patients in IT and IC phase were younger than those in LR and ENH phase. More male patients were presented in the IC and ENH phase. Serum HBV DNA and quantitative HBsAg levels were significantly higher in IT and IC phase than LR phase.

**Table 1:** Demographic Characteristics of Chronic HBV-infected Patients and Healthy Volunteers.

	Healthy volunteers	Chronic HBV-infected patients			
		Immune tolerance phase	Immune clearance phase	Low- replicative phase	HBeAg- negative hepatitis phase
Number	38	20	35	35	34
Age (years)*	48.2 ± 12.4	34.8 ± 6.6^&,$^	35.1 ± 11.0^£,¥^	49.5 ± 10.0^&,£^	49.7 ± 12.1^$,¥^
Male**	22 (78.6)	9 (45%)	28 (82.9)	21 (58.3)	29 (85.3)
ALT (U/L)*	27.1 ± 16.5	25.8 ± 7.80	321.9 ± 272.7	21.4 ± 6.8	313.9 ± 277.7
HBV DNA (log IU/mL)*		7.48 ± 1.42	7.36 ± 1.28	3.52 ± 1.31	6.16 ± 1.55
HbsAg (log IU/mL)*		4.15 ± 0.81	3.47 ± 1.20	2.17 ± 1.14	2.85 ± 0.91

^&,$,£,¥^p < 0.05. *Mean ± SD; **no. (%).

### The frequency of Tregs of peripheral blood increased only in IC and ENH phases and correlated with serum alanine aminotransferase level

**Figure 1:**
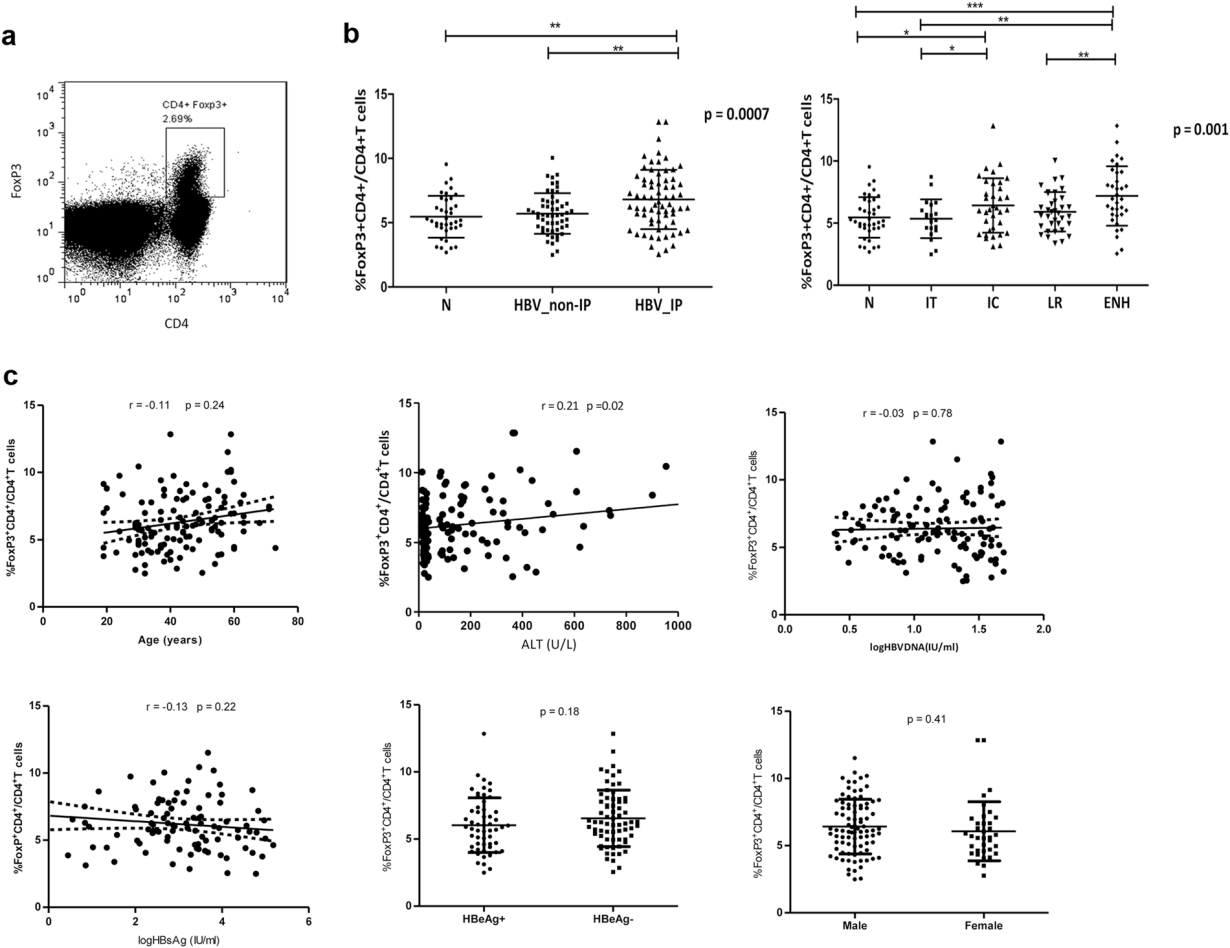
Tregs in peripheral blood of chronic HBV-infected patients and healthy volunteers. (**a**) Direct *ex vivo* flow cytometry staining experiment from a representative chronic HBV patient using antibodies against CD4 and FoxP3. Cells were gated on lymphocyte on the basis of forward and side scatter properties. The isotype-matched antibodies were used as controls. Number indicates the percentage of cells in the indicated region. (**b**) Percentage of Tregs (CD4^+^FoxP3^+^T cells within CD4^+^T cell fraction) of distinct immune phase was determined by FACS staining of PBMC of 124 chronic HBV patients and 38 healthy volunteers. (**c**) Association between circulating Treg frequency and serum HBV DNA load, HBsAg titer, age or ALT levels in chronic HBV patients. IP, inflammatory phase. Error bars denote the mean ± standard deviation. *p < 0.05, **p < 0.01, ***p < 0.001.

The proportion of Tregs in CD4^+^ T cells population of peripheral blood was analyzed using the gate shown in Fig. 1a. A higher percentage of Tregs was detected in chronic HBV infected patients compared with healthy volunteers (6.32 ± 2.08% vs. 5.47 ± 1.63%; p = 0.022). There was variability in the frequency of Tregs among the four phases of patients and healthy volunteers (Fig. 1b). In chronic HBV infected patients with HBeAg, the frequency of Tregs increased significantly in IC phase than in IT phase (6.43 ± 2.19% vs. 5.35 ± 1.56%; p = 0.048). In patients negative for HBeAg, the frequency of Tregs increased significantly in ENH phase than in LR phase (7.19 ± 2.39% vs. 5.92 ± 1.59%; p = 0.007). Both the frequency of Tregs in IC and ENH phase was higher than that of the healthy volunteers (N) (IC vs. N; 6.43 ± 2.19% vs. 5.59 ± 1.50%, p = 0.035, and ENH vs. N; 7.19 ± 2.39% vs. 5.59 ± 1.50%, p < 0.001). However, there was no significant difference in the percentage of Tregs between healthy volunteers and CHB patients in IT and LR phases.

In order to determine the factors that influence the frequency of Tregs of peripheral blood in patients with chronic HBV infection, host and viral characteristics of patients including age, gender, serum ALT, HBV-DNA and HBsAg levels and HBeAg status were analyzed using Spearman’s rank correlation analysis. As shown in Fig. 1c, there was a positive linear correlation between circulating Tregs and serum ALT levels (r = 0.21; p = 0.02). However, the frequency of circulating Tregs did not correlate with age, gender, serum HBV-DNA and HBsAg levels, and HBeAg status (p > 0.05). This suggested that this increase of Tregs was associated with amplitude of liver necroinflammation.

### The increase of Tregs in inflammatory phase of chronic HBV infection were of thymus-origin and expressed higher CCR5, Ki67 and Tim-3 than healthy volunteers

**Figure 2:**
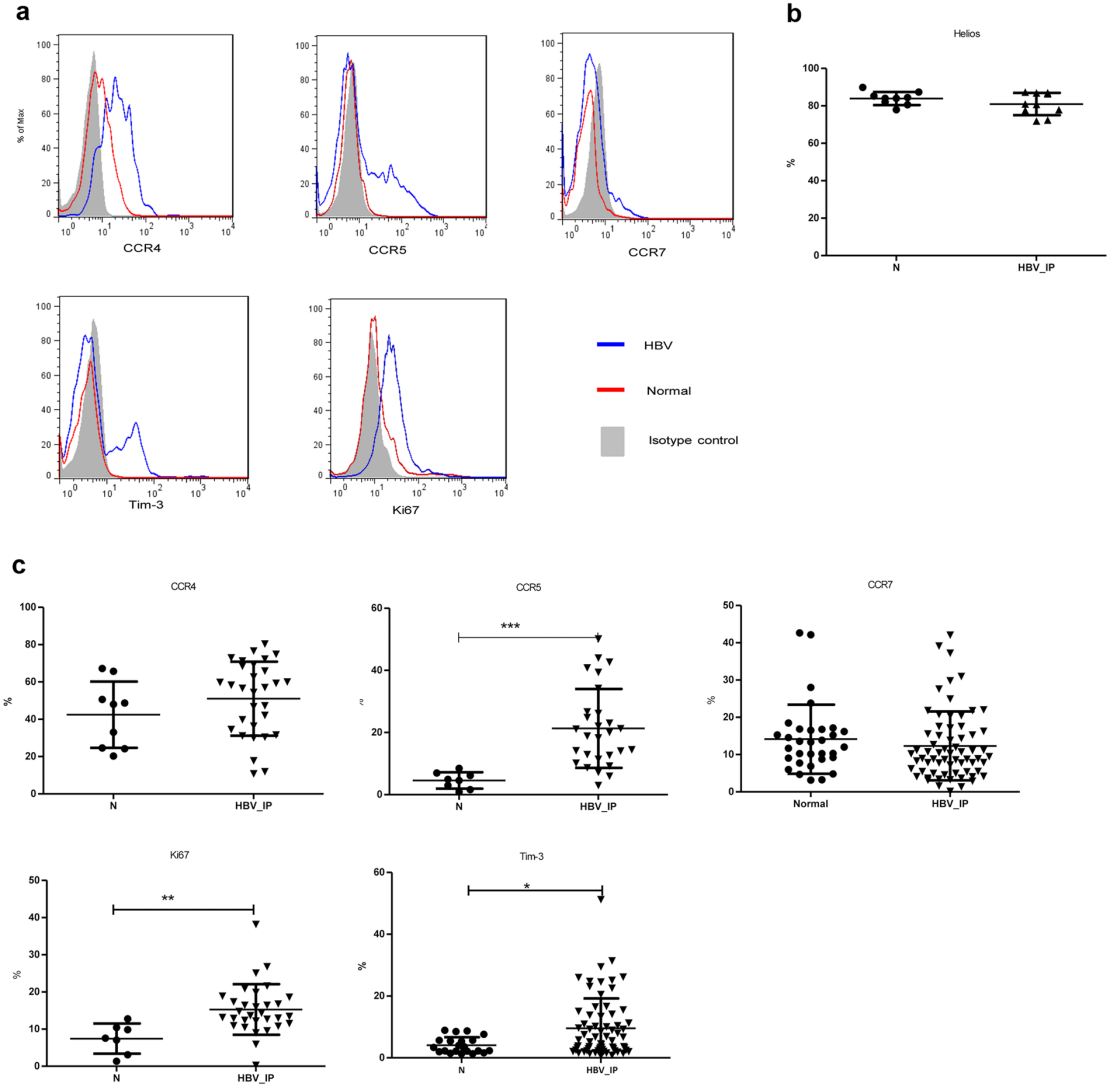
Differences of phenotypic marker expressions on the Tregs in chronic hepatitis B patients and healthy individuals. (**a**) Histograms represented the frequency of CCR4, CCR5, CCR7, Ki67, and Tim-3 expression in Tregs. One representative experiment of gating on CD4^+^FoxP3^+^ population was shown. The isotype-matched antibodies were used as controls. (Gray area represents isotype-matched antibody, blue line represents HBV patient, and red line represents healthy control). (**b**) Frequency of Helios expressing Tregs of chronic HBV-infected patients in inflammatory phases and healthy individuals. (**c**) The proportion of CCR4, CCR5, CCR7, Ki67, and Tim-3-expressing Tregs within the CD4^+^FoxP3^+^T cells was shown. Error bars denote the mean ± standard deviation are presented. *p < 0.05, **p < 0.01, ***p < 0.001.

Further to examine the phenotypically difference on the Tregs, peripheral blood mononuclear cells (PBMCs) from healthy volunteers and chronic HBV infected patients on inflammatory (IC and ENH) phase were analyzed (Fig. 2a).

The increased frequency of Tregs in CHB patients during liver inflammation might arise from the expansion of thymic-derived natural Tregs or the de novo induction from naïve T cells. Helios, a member of the Ikaros transcription factor family, had been demonstrated to be expressed by 100% of CD4^+^CD8^−^Foxp3^+^ thymocytes. Our data showed the proportion of Tregs with positive Helios expression was similar between healthy volunteers and chronic HBV patients (N vs. HBV, 84.0 ± 3.5% vs. 81.0 ± 6.0%; p = 0.36) (Fig. 2b).

Next, we investigated the chemokine receptors for the Treg trafficking and retention in inflammatory site (Fig. 2c). We found that more Tregs in inflammatory phase of chronic HBV infection expressed C-C chemokine receptor 5 (CCR5), which was a proinflammatory chemokine receptor and indicated these Tregs possessed potential to migrate to inflammatory tissue than healthy volunteers (N vs. HBV, 4.6 ± 2.6% vs. 21.4 ± 12.7%; p < 0.001). However, the percentage of C-C chemokine receptor 4 (CCR4) and C-C chemokine receptor 7 (CCR7) expressing Tregs was similar (CCR4, N vs. HBV, 42.5 ± 17.8% vs. 51.0 ± 19.8%; p = 0.22; CCR7, N vs. HBV, 14.1 ± 9.3% vs. 12.3 ± 9.2%; p = 0.19). In addition, a higher percentage of Tregs of CHB patients expressed Ki67, a protein highly expressed in proliferating cells, than the healthy volunteers (N vs. HBV, 7.4 ± 4.1% vs. 15.3 ± 6.8%; p = 0.002). T-cell immunoglobin mucin 3 (Tim-3) had previously been demonstrated as a marker for a subgroup of Tregs that exerted more robust immunosuppressive functions with the tendency to accumulate in the inflammed tissue^21,22^. Here, we found more CHB patients had a higher proportion of Tim-3 expressing Tregs than healthy volunteers (N vs. HBV, 4.1 ± 2.6% vs. 9.6 ± 9.7%; p = 0.01).

All these data implied that the increased Tregs in patients with chronic HBV infection in IC and ENH phases were possibly from proliferation of the thymus-origin natural Tregs and with potential to migrate to inflamed tissue and more immunosuppressive ability.

### Accumulation of Tregs into the liver in patients with chronic HBV infection

**Figure 3:**
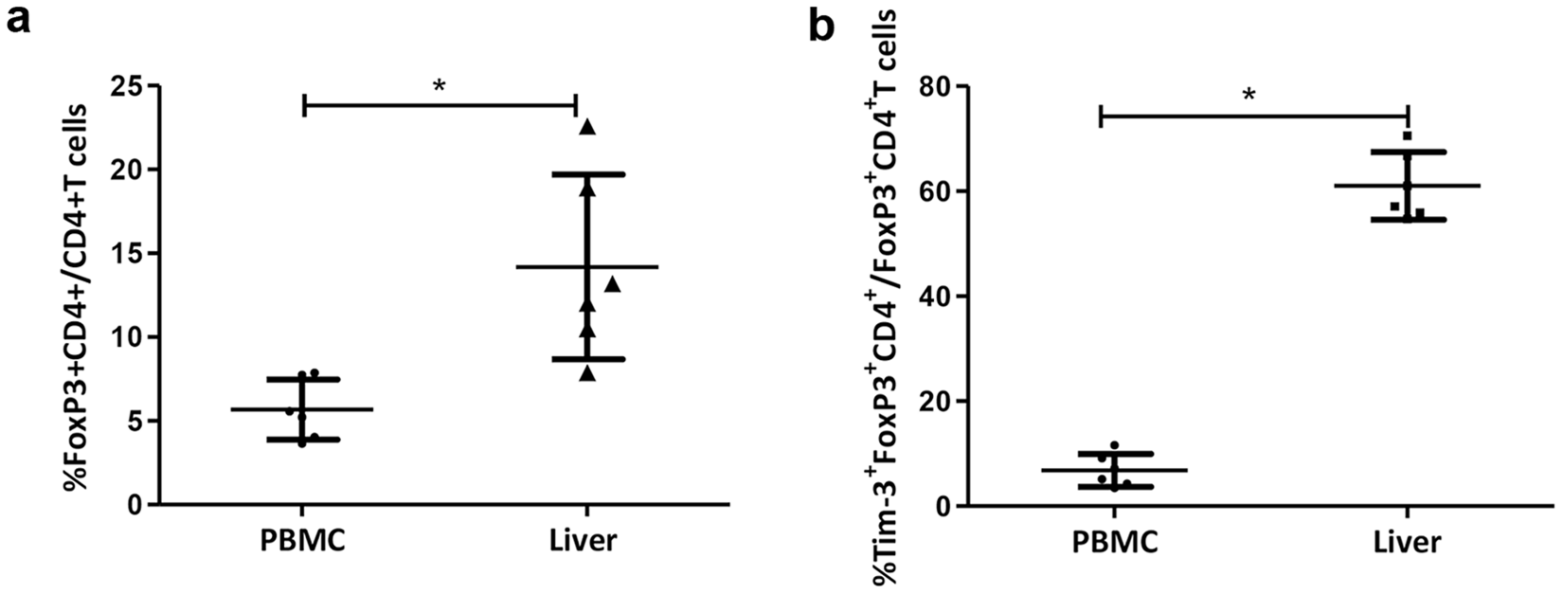
Accumulation of Tregs inside liver tissue of chronic hepatitis B patients. (**a**) The frequency of Tregs in liver infiltrating lymphocytes and peripheral blood were detected using flow cytometry analysis. (**b**) The proportion of Tim-3 expression of Tregs in liver infiltrating lymphocytes and peripheral blood. Error bars denote the mean ± standard deviation; *p < 0.05.

The increased frequency of Tregs with higher Ki67 and Tim-3 expression in CHB patients with elevated ALT levels raised the possibility that the inflamed liver might be the site where tissue-prone Tregs accumulated (Fig. 2c). In order to investigate whether Tregs were accumulated in the liver for limiting local inflammation, liver infiltrating lymphocytes were isolated from liver tissue of six CHB patients with serum ALT > 2 upper limit of normal receiving liver biopsy. In comparison with peripheral blood, the proportion of Tregs in total liver infiltrating lymphocytes of liver tissue was significantly increased than those in peripheral blood (14.2 ± 5.5% vs. 5.7 ± 1.8%; p = 0.03) (Fig. 3a). Furthermore, the liver-infiltrating Tregs expressed even higher proportion of Tim-3 than Tregs of the peripheral blood (61.0 ± 7.2% vs. 6.8 ± 3.1%; p = 0.03) (Fig. 3b).

### Memory Tregs, not rTregs, increase during IC and ENH phases

**Figure 4:**
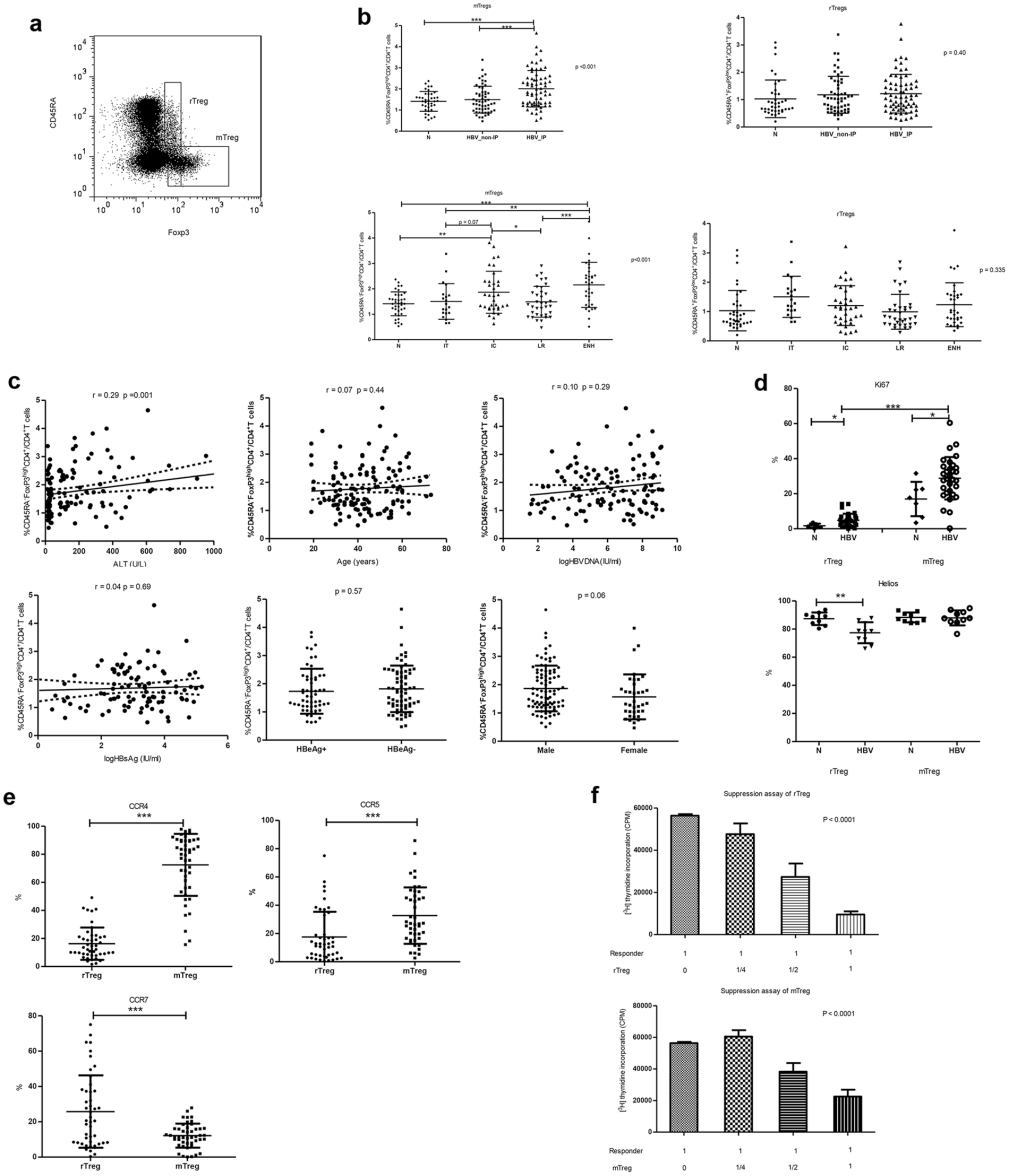
Memory Tregs, but not rTregs, increased during the IC and ENH phases and possessed suppressive ability and with higher pro-inflammatory chemokine receptors expression. (**a**) Direct *ex vivo* flow cytometry staining experiment from a representative chronic HBV patient using antibodies against CD4, CD45RA and FoxP3. Cells were gated on CD4^+^T cells. The isotype-matched antibodies were used as controls. (**b**) The percentages of rTregs and mTregs of PBMC were determined by FACS staining of 124 chronic HBV patients and 38 healthy volunteers. (**c**) Association between circulating mTreg frequency and serum HBV DNA load, HBsAg titer, age or ALT levels in patients with CHB. (**d**) Frequency of Ki67 and Helios expressing mTregs and rTregs of chronic HBV-infected patients in inflammatory phase and healthy individuals. (**e**) The proportion of chemokine receptor CCR4, CCR5 and CCR7-expressing mTregs and rTregs withon the CD4^+^FoxP3^+^ T cell in chronic HBV-infected patients was shown. (**f**) rTregs and mTregs inhibit T cell proliferation in a dose-dependent manner. CD25-depleted CD4^+^T cells were co-cultured with rTregs or mTregs in a dose dependent manner (CD25-depleted CD4^+^T cells: rTregs or mTregs: 1:0; 1:1/4; 1:1/2; 1:1). The proliferation of the cells was determined by the incorporation of [3 H] thymidine after stimulated for 4 days with anti-CD3/CD28 mAbs. Statistical analysis for rTreg and mTreg was performed on the logarithmic transformation of the counts per minute with a random intercept and a random slope using Linear Mixed-Effects model. Estimated population average profile simple linear regression of rTreg: intercept = 11.12 (p < 0.0001) and mean slope = −1.86 (p < 0.0001). Estimated population average profile simple linear regression of mTreg: intercept = 11.10 (P < 0.0001) and mean slope = −1.03 (P = 0.0001). Error bars denote the mean ± standard deviation. *p < 0.05, **p < 0.01, ***p < 0.001.

Subsequently, we studied the subgroups of Tregs including mTregs and rTregs in patients with chronic HBV infection (Fig. 4a). It was found the frequency of rTregs was similar among different immune phases. However, the frequency of mTregs in IC and ENH phase increased significantly when compared with that of IT and LR phases and healthy individuals (N, IT, IC, LR, and ENH; 1.4 ± 0.5%, 1.5 ± 0.7%, 1.9 ± 0.8%, 1.5 ± 0.6%, and 2.2 ± 0.9%, respectively; p < 0.001) (Fig. 4b). To determine the factors influencing the frequency of mTregs, host and viral parameters were analyzed by spearman rank correlation analysis. As shown in Fig. 4c, mTreg frequency was only correlated with serum ALT levels (r = 0.29; p = 0.001) but not with age, gender, serum HBV-DNA and HBsAg levels, and HBeAg status (P > 0.05).

Furthermore, we analyzed the phenotype of these mTregs. As shown in Fig. 4d, the Ki67 expression on the mTregs was significantly higher in patients with CHB than healthy volunteers (28.8 ± 12.1% vs. 17.0 ± 9.8%; p = 0.02). Interestingly, the proportion of mTregs with positive Helios expression, a marker of thymic Tregs, was similar in CHB patients and health volunteers (HBV vs. N, 88.0 ± 5.3% vs. 88.3 ± 3.5%; p = 0.78).

As for the chemokine receptor expression on mTregs and rTregs in CHB patients, as shown in Fig. 4e, a higher proportion of mTregs expressed CCR4 and CCR5 than rTregs (CCR4, mTreg vs. rTregs, 72.5 ± 22.1% vs. 16.3 ± 11.5%, p < 0.001; CCR5, mTreg vs. rTregs, 32.7 ± 20.0% vs. 17.5 ± 17.8%, p < 0.001). In addition, there was a lower proportion of mTregs with CCR7 expression than rTregs (12.1 ± 6.8% vs. 25.8 ± 20.5%, p < 0.001). These data suggested that the mTreg was the main subgroup of Tregs increased in chronic HBV infection during liver inflammation. Furthermore, mTregs were mainly thymic origin and were highly replicative and possessed prominent potential to migrate to inflammatory site.

### Both the mTregs and rTregs possessed similar suppressive ability and inhibited proliferation of CD4^+^T cells

In order to demonstrate the suppressive ability of these Tregs from CHB patients, we sorted these subgroups of Tregs and evaluated their suppression ability. As shown in Fig. 4f, both rTregs and mTregs could inhibit the CD4^+^CD25^−^ T cell proliferation in a dose-dependent manner.

### The increased mTregs in chronic hepatitis B were related to galectin-9/Tim-3 interaction through the increased serum levels of galectin-9

**Figure 5:**
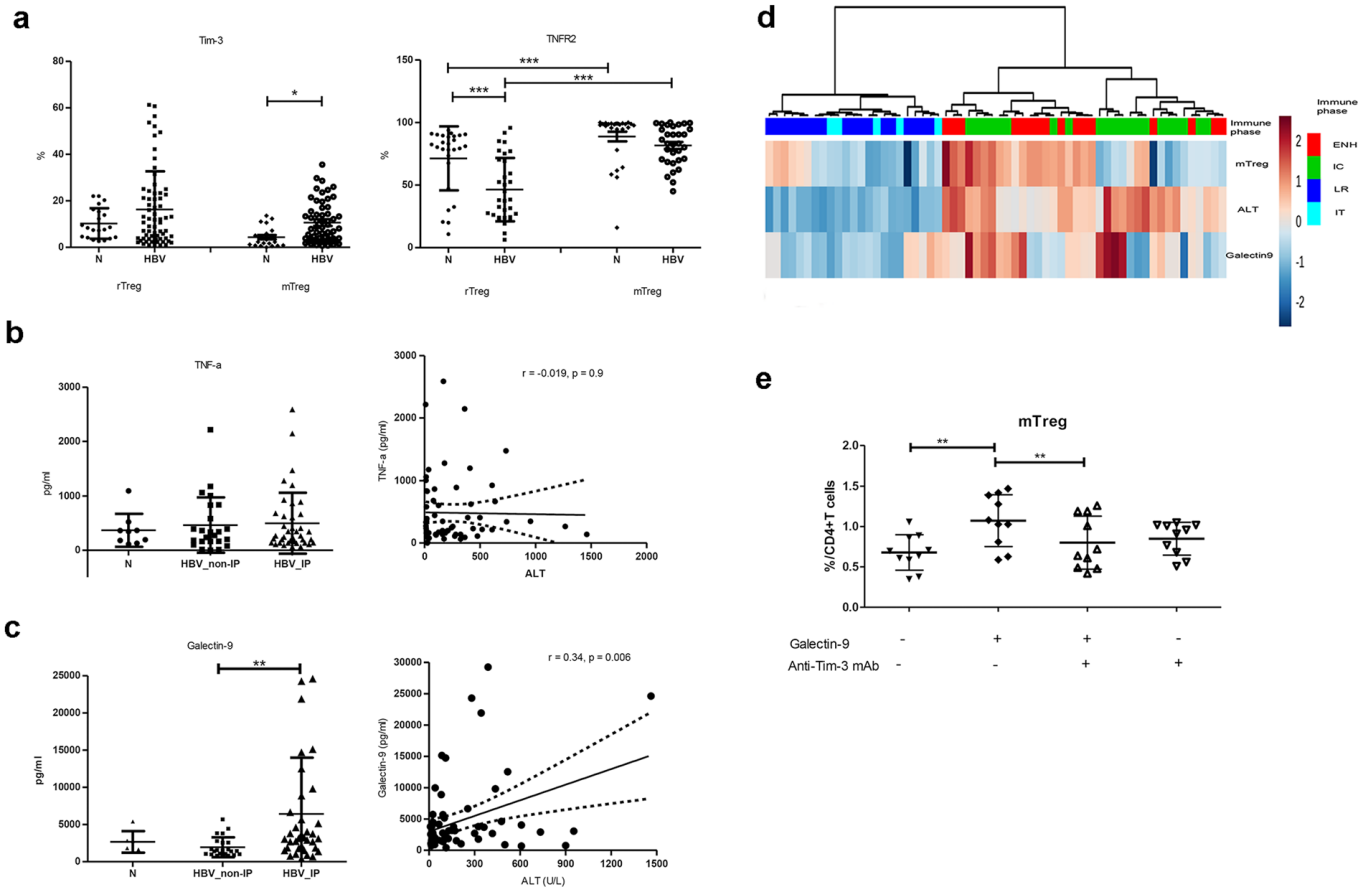
Galectin-9, but not TNF-α, was increased in the plasma of chronic HBV patients in the IC and ENH phase, and was associated with expansion of mTregs. (**a**) Proportion of Tim-3 and TNFR2-expressing rTregs and mTregs of CHB patients in IC and ENH phase and healthy volunteers. (**b**) Serum levels of TNF-α of healthy volunteers and HBV patients. (**c**) Serum levels of galectin-9 of healthy volunteers and HBV patients. (**d**) Unsupervised hierarchical clustering using Euclidean distance. Dendrogram displays similarities between clusters. Increasing color intensity (blue- >red) corresponds to increasing mTreg frequency, serum ALT or galectin-9 levels in CHB patients. (**e**) The addition of galectin-9, anti-Tim-3 mAb, and galectin-9 plus anti-Tim-3 mAb at day 0 of culture with purified CD4^+^T cells. Anti-Tim-3 mA blocked the induction mTregs expansion. Error bars denote the mean ± standard deviation. *p < 0.05; **p < 0.01; ***p < 0.001.

Next, we determined which mediators could cause the expansion of mTregs. There were two interesting molecule-interactions that involved in the Treg expansion. One was the tumor necrosis factor-α (TNF-α)/tumor necrosis factor receptor type 2 (TNFR2) interaction and another was the Tim-3/galectin-9 interaction. The expression of TNFR2 was higher in mTregs when compared with rTregs in both HBV-infected patients and healthy volunteers (mTreg vs. rTreg in HBV, 81.6 ± 15.6% vs. 46.4 ± 25.3%; p < 0.0001; mTreg vs. rTreg in normal volunteers, 88.8 ± 20.2% vs. 71.4 ± 25.6%; p < 0.0001), though there was no difference between HBV-infected patients and healthy volunteers in terms of the percentage of TNFR2 expressed mTregs (Fig. 5a). We then analyzed the serum TNF-α levels in CHB patients and healthy individuals. The serum TNF-α levels were similar among CHB patients and healthy volunteers and were not related to serum ALT levels in CHB patients (r = −0.019, p = 0.9) (Fig. 5b).

In previous results described above, Tim-3 expression was up-regulated on Tregs and also on mTregs of CHB patients (HBV vs. N, 10.63 ± 12.21% vs. 4.38 ± 4.26%; p < 0.05). However, this difference was not found in rTregs (Fig. 5a). As for the galectin-9, the serum levels of galectin-9 in chronic HBV patients with normal serum ALT were similar with that in healthy volunteers but increased in HBV patients with elevated ALT levels. In addition, the serum levels of galectin-9 in patients with chronic HBV infection were correlated positively with serum ALT levels (r = 0.34, p = 0.006) (Fig. 5c). These findings were concisely displayed via hierarchical clustering by Euclidean distance (Fig. 5d). IC and ENH phase clustered together, with IT and LR phase forming a separate cluster. Clustering based on similarity of serum ALT, galectin-9 and mTreg frequency, it was showed that a higher frequency of mTreg was concordant with high serum ALT and galectin-9 levels. These data indicated galectin-9 play a role in the increment of mTregs in CHB patients in inflammatory phase.

Next we explore whether the mTregs could be expanded through galectin-9/Tim-3 interaction. As shown in Fig. 5e, we found galecitn-9 could expand the mTregs *in vitro* and this increment could be reversed by anti-Tim-3 mAbs. Taken together, the increased serum levels of galecin-9 during HBV infection contributed to the expansion of mTregs through the galectin-9/Tim-3 interaction.

## Discussion

The role of Tregs in chronic HBV infection, especially for their relationship to the chronicity of HBV infection, is controversial^11,19,23^. However, all the evidences are mainly derived from CHB patients with liver inflammation. By investigating the Tregs in different phases, including IT, IC, LR and ENH phases, it could possibly decipher the real role of Tregs in chronic HBV infection. Here, we show the Tregs are only increased in the IC and ENH phases but not in the IT and LR phases. In addition, it is the mTregs rather than rTregs that are responsible for this increase in Tregs. We also show these mTregs with potent suppressive ability would accumulate in the inflamed liver and could be expanded through galectin-9/Tim-3 interaction. Furthermore, the increased serum levels of galectin-9 are associated with liver inflammation (ALT levels). Therefore, we conclude that it is the mTregs that are responsible for the increased Tregs in chronic HBV infection. This increase of mTregs is only correlated with liver inflammation and possibly through galectin-9/Tim-3 interaction.

The functional delineation of Foxp3^+^CD4^+^T cells into rTregs and mTregs based on CD45RA and FoxP3 expression^16,17^ had lead to the observations that mTregs were main suppressors in chronic inflammatory disease, sarcoidosis, liver cirrhosis and cancer^17,19,24,25^. The importance of these mTregs in inflammation is not only they are potent suppressors but also they possess the pro-inflammatory chemokine receptors and migrate into inflamed tissues^19,26^. In our analysis, the increased frequency of Tregs in patients with CHB is mainly due to the increased mTregs but not the rTregs. Furthermore, this increase of mTregs is only found in patients in the IC and ENH phases but not in the IT and LR phases. In other words, this increase in the mTregs is correlated with liver inflammation that only found in the phases of IC and ENH, so called inflammatory phase of chronic HBV infection^6^. This inflammation related increase of Tregs, especially the mTregs, could also be deduced from the results that the frequency of mTregs is only correlated with the serum level of ALT but not serum HBV-DNA levels, HBsAg titers, age, gender and HBeAg status. However, in previous reports, frequency of Tregs was shown to be correlated with HBeAg status^11^, serum HBV viral titer^13^ but not the serum ALT levels^11,13^. The possible explanation could be the different patient population enrolled in different studies that cause these discrepancies. In the present study, patients in four different phases were enrolled based on well-defined definition. However, in other studies, only patients with abnormal ALT were enrolled. Recently, an elegant study focused on the T cell functions in CHB patients of different phases also claimed the increase of Tregs was only in IC and ENH phases, compatible to our results^27^. Therefore, the increased Tregs only found in the inflammatory phases of chronic HBV infection but not in the non-ifnlammatory phase chronic HBV infection have implied the increase of Tregs in chronic HBV infection is only a protective immune response for the liver inflammation but not for the chronicity of HBV infection. Interestingly, recent publication claimed the myeloid-derived suppressor cells increased in the immune tolerance phase of patients with chronic HBV infection and possibly was the cause of chronicity of HBV infection^28^. This observation reinforces the notion that Tregs are not the cause of chronicity of HBV infection but are responsible for the protection against immune-mediated pathology of liver.

For regulating the immune responses, Tregs would migrate to the inflamed tissue for inhibiting the ongoing inflammation^29^. In patients with CHB, the Tregs also showed the tissue-migration tendency and accumulated in the inflamed liver as shown in this study and in previous report^13^. In previous reports, mTregs were the Tregs that could migrate to the inflamed tissue for suppression of immune responses^30–32^. In the present study, these mTregs also express higher levels of pro-inflammatory chemokine receptors like CCR4 and CCR5 but less lymph-node homing chemokine receptor like CCR7. These observations are similar to the mTregs in patients with colon cancer^19^ and indicate it is a general phenomenon that these mTregs are responsible for suppressing the immune responses in inflamed tissue.

Another interesting issue is why these mTregs could be expanded and associated with liver inflammation. In our previous report, we had shown that the TNF-α could augment proliferation of mTregs through TNF-α/TNFR2 interaction in patients with hepatoma and colon cancer^18^. Though the surface expression of TNFR2 is higher in mTreg than that of rTreg in patients with CHB, we do not find significant increased serum levels of TNF-α in CHB patients compared with the healthy volunteers. Therefore, the possibility that mTregs a expanded by TNF-α in patients with CHB is not likely.

On the other hand, our results show the increased serum levels of galectin-9 are associated with the increase serum levels of ALT. It is not a surprising result. It had been reported that increased serum levels of galectin-9 were associated with liver inflammation in patients with CHB^33^ and chronic hepatitis C^34^. In addition, the galectin-9 could also expand the Tregs as well^34,35^. In present study, we demonstrate the Tim-3 expression is significantly higher on the mTregs when compared with rTregs. Furthermore, we show galectin-9 could expand the mTregs through galectin-9/Tim-3 interaction. In addition, we also show the relationship among serum levels of ALT, serum levels of galectin-9 and mTregs by hierarchical clustering analysis. Therefore, it is reasonable to claim the increased serum levels of galectin-9 that associated with liver inflammation could in turn expand the mTregs that migrate to the liver to limit the liver immunopathology in patients with CHB.

Another issue is the origin of these mTregs. In our data, we have shown these mTregs had higher Ki-67 expression than rTregs and healthy volunteers. Furthermore, the Helios expression is similar in mTregs between patients with CHB and healthy volunteers. Helios is a member of the Ikaros transcription factor family and is deemed as a marker for the natural Treg population^36,37^. Therefore, it is more likely that these expanded mTregs are from thymic-derived Treg population rather than HBV-specific Tregs. This observation further re-enforce the idea that these Tregs are not the cause of chronicity of HBV infection but for the protection against liver immune-pathology. However, this observation does not exclude the role of HBV-specific Tregs in the chronicity of HBV infection. It is still possible that these HBV-specific Tregs are a kind of induced Tregs and locate in the Helio-negative Tregs in these patients^38^.

In summary, increased mTregs are found in patients of CHB with inflammatory phase (IC and ENH) but not with non-inflammatory phase (IT and LC) and would accumulate in the inflamed liver. Galectin-9, which is associated with liver inflammation through galectin-9/Tim-3 interaction, contributes to the expansion of the mTregs that are mainly originated from natural Tregs. Therefore, this expansion of mTregs only reflects as an immune regulatory mechanism to limit the on-going liver damages rather than the cause of chronicity of HBV infection.

## Materials and Methods

### Study population

We prospectively enrolled 124 patients with chronic HBV infection and 38 healthy controls into the study. None of the patients had received antiviral therapy before enrollment in this study and they were divided into four immune phases according to natural course of chronic HBV infection. The first was IT phase, where HBeAg-positive patients had high viral loads (more than 2 × 10^6^ IU/mL) but normal serum ALT levels. The second was IC phase, which was ALT flare (>2 × upper limit of normal) in HBeAg-positive patients^1,2^. After HBeAg seroconversion, patient entered the third LR phase with sustained normalization of ALT and low viral load (70~80% patients had serum HBV DNA less than 2 × 10^3^ IU/ml)^3,39,40^. The fourth phase was ENH phase, where 10~20% of these HBeAg-negative patients encountered ALT flare (>2 × upper limit of normal) and high serum HBV-DNA (>2 × 10^3^ IU/ml)^41–43^. Patients with concurrent hepatitis C virus infection, toxic hepatitis, autoimmune hepatitis, primary biliary cirrhosis, or Wilson’s disease were excluded from this study. Patients with evidence of decompensated liver cirrhosis, chronic alcohol abuse, and psychiatric problems were also excluded. Heparinized peripheral blood samples were obtained from the participants. Liver biopsy specimens were obtained by needle puncture. Informed consents were given to all participants before blood donation. The study was performed in accordance with the ethical guidelines of the International Conference on Harmonisation for Good Clinical Practice and has been approved by the Institutional Review Board of Chang Gung Memorial Hospital (99-0489B, 100-1894B & 102-1058B). Informed consent was obtained from all enrolled patients and healthy volumteers.

### Isolation of PBMCs and flow cytometric analysis

PBMCs were isolated from heparinized blood samples by Ficoll-Paque plus (Amersham, Uppsala, Sweden) density gradient. After isolation, cells were washed two times with RPMI-1640 (Gibco, Auckland, N.Z.) and prepared for further study. The surface and intracellular stainings were performed using the following fluorochrome-conjugated antibodies: anti-CD4-PerCP, anti-CD45RA-FITC, anti-CCR4-PE, anti-CCR5-PE, anti-CCR7-PE, anti-Ki67-PE (BD Pharmingen, San Diego, CA), anti-Tim3-PE, anti-TNFR2-PE (R&D Systems, Minneapolis, MN), anti-Helios-PE, anti-FoxP3-APC (eBioscience, San Diego, CA). For intracellular staining, cells were fixed and permeabilized using the Human FoxP3 Buffer Set (eBiosciences, San Diego, CA) according to the manufacturer’s instructions. Isotype-matched control antibodies were used to define the positive staining populations. Stained cells were acquired and analyzed using a FACSAria cytometer (Becton Dickinson, San Jose, CA, USA) with FACSDiVa software (BD Biosciences).

Liver biopsy specimen was obtained from six chronic HBV infected patients and was chopped and incubated with collagenase-D (0.1%) (Gibco, Waltham, USA) in RPMI-1640 with 10% fetal bovine serum (Gibco, Grand Island, NY). After incubate and filtered through nylon mesh, cells were suspended in RPMI-1640 and then stained with anti-CD4-PerCP, anti-CD45RA-FITC, anti-Tim3-PE and anti-Foxp3-APC.

### Isolation of Tregs

Fresh isolated PBMCs were stained with anti-CD4-PerCP, anti-CD45RA-FITC and anti-CD25-PE antibodies (BD Pharmingen, San Diego, CA). Then CD45RA^+^CD25^low^CD4^+^T cells (rTregs) and CD45RA^−^CD25^high^CD4^+^T cells (mTregs mTregs) were isolated from PBMCs using a FACSAria (Becton Dickinson) flow cytometer according the manufacturer’s instruction. CD4^+^CD25^−^ T cells were also collected for further use as well. The purity of sorting was >99%.

### Suppression assay of Tregs

Briefly, CD45RA^+^CD25^low^CD4^+^T cells (rTreg), CD45RA^−^CD25^high^CD4^+^T cells (mTreg) and CD25^−^CD4^+^(non-Tregs) T cells were isolated from PBMC by FACSAria (BD Bioscience).

Freshly isolated CD4^+^CD25^−^T cells were used as responder cells. Inhibition of proliferation of CD4^+^CD25^−^T cells was tested by adding 25% (5,000 cells), 50% (10,000 cells) and 100% (20,000 cells) rTregs or mTregs to 2 × 10^4^ CD4^+^CD25^−^ T cells in RPMI-1640 medium containing 10% fetal bovine serum. Cells were stimulated with anti-CD3/CD28 (R&D systems, Minneapolis, USA) coated beads (Invetrogen) for 96hrs. After incubation, the cells were pulsed with 1 μCi/well [3 H] thymidine (Perkin Elmer, Boston, USA) and harvested 16 hours later by scintillation counting.

### Measurement of serum galectin-9 and TNF-α

Serum levels of galectin-9 of 60 chronic HBV infected patients and 10 healthy controls were measured using a Galectin-9 ELISA Kit (R&D Systems, Minneapolis, USA). 96-well plates were coated with mouse anti-human Galectin-9 in PBS, and blocked by Reagent Diluent. 100 uL of serum or standards in Reagent Diluent, or an appropriate diluent, was added per well and incubated for 2 hours. Sample was detected using biotinylated goat anti-human Galectin-9. Quantification was performed using Streptavidin-conjugated horseradish peroxidase. Substrate Solution was used for color development, and the optical density was determined with a microplate reader at 450 nm. Serum levels of TNF-α of 64 chronic HBV infected patients and 9 healthy controls were measured using a BD Cytometric Bead Array (Becton, Dickinson, and company, San Jose, CA, USA) as manufacturer’s instruction.

### *In vitro* expansion assay

Purified CD4^+^T cells isolated from PBMCs of 10 patients by Human CD4^+^T cell Enrichment kit (Stemcell Technologies, Vancouver, BC, Canada) were cultured in triplicate in a concentration of 4 × 10^5^ cells per well in 200 ul RPMI-1640 containing 10% fetal bovine serum. The cells were stimulated with or without 2 ug/ml galectin-9 (PROSPEC, East Brunswick, USA). The anti-Tim-3 antibodies (5 ug/ml) (Biolegend, San Diego, CA) were added into wells before galectin-9 stimulation or not. The cells not stimulated with galectin-9 or anti-Tim-3 mAb were used as control. Then the cells were harvested after 5 days and stained CD4-PerCP, CD45RA-FITC and Foxp3-APC. After staining, the cells were analyzed with a four-color cytometer (FACS-caliber; CELLQuest Pro software, Beckton Dickinson).

### Statistical Analysis

The continuous variables were expressed as mean ± standard deviation values. Two-tailed Student’s unpaired t-test or Mann-Whitney U-test was used to evaluate the difference between the two groups where appropriate. Differences between groups of categorical variables were analyzed using chi-square test. One-way analysis of variance with post Hoc comparison Fisher’s least significant difference was performed to evaluate the difference between multiple groups. Correlation between the Tregs and clinical factors were determined by spearman’s rank correlation analysis. Statistical analysis for suppression assay was performed using Linear Mixed-Effect model. Comparison of the expression of Tregs in PBMC and liver from the same individual and analysis of the *in vitro* expansion assay results were performed using the Wilcoxon signed rank sum test. Hierarchical clustering was performed using Euclidean distance to evaluate the similarity of each factor to one another. A p value < 0.05 was considered statistically significant. The statistical analyses were performed using the SPSS ver. 12.0 software (SPSS Inc., Chicago, USA).
